# Abortion‐related training in maternal‐fetal medicine fellowship: A national survey of program directors and fellows

**DOI:** 10.1002/pmf2.70239

**Published:** 2026-01-21

**Authors:** CeCe Cheng, John J. Byrne, Jennifer L. Kerns, Patrick S. Ramsey, Ashish Premkumar

**Affiliations:** ^1^ Division of Maternal‐Fetal Medicine Department of Obstetrics & Gynecology University of Washington Seattle Washington USA; ^2^ Division of Maternal‐Fetal Medicine Department of Obstetrics & Gynecology University of Texas Health Science Center at San Antonio San Antonio Texas USA; ^3^ Division of Complex Family Planning Department of Obstetrics, Gynecology and Reproductive Sciences University of California San Francisco California USA; ^4^ Division of Maternal‐Fetal Medicine Department of Obstetrics & Gynecology, University of Chicago Chicago Illinois USA

**Keywords:** abortion care, fellowship education, maternal‐fetal medicine, program director, training

## Abstract

**Introduction:**

Policies that restrict abortion access not only create unnecessary and dangerous barriers to care for patients but also disrupt evidence‐based medical training in residency and subspecialty training. Previous studies indicate that exposure to abortion procedures during residency is directly linked to interest in pursuing abortion‐related training in maternal‐fetal medicine (MFM) fellowship and subsequent clinical practice. However, limited data exist regarding whether MFM trainees and program directors (PDs) share similar views on abortion‐related training objectives. This study aims to identify differences in perspectives between MFM fellows and PDs regarding the importance of abortion training during fellowship. Our secondary objective is to evaluate whether perspectives differ based on the state‐based abortion restrictions.

**Methods:**

A web‐based cross‐sectional survey was conducted in June 2022, targeting MFM PDs and fellows to evaluate the importance participants placed on access and exposure to training in procedures related to abortion. These procedures included dilation and curettage (D&C), dilation and evacuation (D&E), induction termination of fetuses with cardiac activity (i.e., induction termination), and induction of fetal asystole (i.e., feticidal injection) during fellowship. Additionally, the survey explored attitudes regarding the importance of specific abortion and reproductive health‐related skills post‐fellowship. All attitudes were measured on a continuous sliding scale from 0 to 100, where 0 indicates “not at all important” and 100 signifies “very important.”

**Results:**

Our findings indicate that fellows in abortion‐restrictive states placed lower importance on most abortion‐related training during fellowship compared to PDs. In abortion‐protective states, there were no differences in importance rated by PDs and fellows regarding abortion‐related training and competency in these skills post‐fellowship. Fellows in abortion‐protective states placed significantly more importance on all skills compared to fellows in abortion‐restrictive states. At the same time, there was no difference between PDs in abortion‐protective or abortion‐restrictive states.

**Conclusion:**

Our findings show that the changing importance of abortion‐related training in this subspecialty is creating both a regional and generational divide in perceptions of what falls within the scope of MFM practice.

## 1 INTRODUCTION

The *Dobbs v Jackson Women's Health Organization* (*Dobbs*) decision in June 2022 marked a turning point in health care in the United States for patients and practitioners alike [1]. Nearly half of the US states subsequently banned or severely restricted abortion access [2]. Policies restricting abortion access not only create unnecessary and dangerous barriers to care for patients but also interfere with evidence‐based medical training and practice. Recent projections estimate that almost half of all Obstetrics and Gynecology (OB/GYN) residents will complete training in abortion restrictive states [3]. The degree of exposure to abortion procedures in residency directly correlates with interest in continuing abortion‐related training in maternal‐fetal medicine (MFM) fellowship and subsequent practice [4, 5, 6]. However, scant data are available to assess whether MFM trainees and program directors (PDs) share the same perspectives on abortion‐related training goals.

We build on our prior work, looking at regional differences in MFM fellows’ perspectives, by focusing on the different perspectives of MFM trainees and PDs on abortion‐related training and experience during MFM fellowship here [4]. Our secondary objective was to identify regional variations, specifically comparing the perspectives of those in fellowships located in abortion‐restrictive states versus those in abortion‐protective states. We hypothesize that MFM fellows would be more motivated to pursue and continue abortion‐related training during fellowship compared to PDs, given the current tumultuous political climate and the increased need for access to abortion care nationwide.

## 2 MATERIALS AND METHODS

We conducted a web‐based, cross‐sectional survey of 98 MFM PDs and 413 MFM fellows at Accreditation Council for Graduate Medical Education (ACGME) approved MFM fellowships between June 6 and 30, 2022. Survey links were sent to each PD and fellow utilizing emails on the Society for Maternal‐Fetal Medicine (SMFM) fellowship program directory and a fellow listserv provided by the Fellowship Affairs Committee of SMFM, respectively. Basic demographic information was collected, including self‐identified race and ethnicity, fellowship location (e.g., state), whether an associated complex family planning (CFP) fellowship was present, and perspectives on support and engagement in abortion advocacy. All survey participants were asked questions about the importance they place on access to and exposure to training in dilation and curettage (D&C), dilation and evacuation (D&E), induction termination of fetuses with cardiac activity (i.e., “induction termination”), and induction of fetal asystole (i.e., feticidal injection) during fellowship. Additionally, we assessed attitudes regarding the importance of specific skills after fellowship, such as the ability to perform abortions independently, induce fetal asystole, work collaboratively with CFP subspecialists, and understand the local legal atmosphere to provide comprehensive reproductive services.

We assessed all attitudes on a continuous sliding scale from 0 to 100, with 0 indicating not at all important, 50 indicating neutral, and 100 indicating very important. Only complete surveys were included in the study. If any individual question was unanswered, the respondent was excluded from the analysis of that question. We did not design the study to examine causal associations; therefore, we did not have a prespecified primary outcome, power calculation, and effect‐size framework. A bivariate analysis was completed comparing all PD responses to MFM fellow responses. A secondary analysis was performed comparing PDs to MFM fellows in abortion‐restrictive versus abortion‐protective states as defined by the Center for Reproductive Health at the time of survey administration [7]. We chose to group states and territories with expanded, protected, and not protected legislation regarding abortion care as abortion‐protective. States defined as not protected were included as abortion‐protective because, when compared with abortion‐restrictive regions, the legislation in place was not as restrictive. We grouped states and territories with hostile or illegal legislation for abortion access together as abortion‐restrictive states. Additional analyses were performed comparing PDs in abortion‐restrictive versus abortion‐protective states, as well as comparing fellows in abortion‐restrictive versus abortion‐protective states [4].

To assess normality, the Shapiro–Wilk test was conducted. Appropriate statistical tests, including Pearson's chi‐squared (χ^2^) test, Fisher's exact test, the Mann–Whitney *U*‐test, and *t*‐tests, were utilized. Categorical variables were presented as frequencies and percentages, while continuous variables were summarized with medians and interquartile ranges. *p* values were corrected for multiple comparisons using the Benjamini–Hochberg procedure to control the false discovery rate at 0.05. All *p* values reported are the adjusted *p* values. A sensitivity analysis excluding unprotected states from the abortion‐protective category was performed and did not result in significant differences (not displayed). All statistical analyses were performed using Prism version 9 (GraphPad Software) and STATA version 19 software (StataCorp LLC). This study was approved by the University of Texas Health Science Center at San Antonio's Institutional Review Board (protocol number 20220276EX).

## 3 RESULTS

Of the 98 PDs and 413 MFM fellows invited to participate in this survey, 61% (60/98) and 46% (190/413) responded, respectively. Fewer PDs identified as female than MFM fellows (60% vs. 79.5%), while other demographic characteristics were similar between the two groups (Table 1). A similar proportion of respondents from abortion‐restrictive and abortion‐protective states was observed, as well as when divided by region. No differences were found in affiliation with CFP fellowships between PDs and fellows. Seventy‐five percent of MFM faculty reported personal involvement in abortion advocacy, compared to 63.2% of fellows who viewed their faculty as engaged in this advocacy. Although MFM faculty reported providing substantial support for fellow advocacy, MFM fellows perceived less support (100 [98.3, 100] vs. 98 [75, 100], Table 1).

**TABLE 1:** Demographics of PDs versus fellows.

	PDs (*n* = 60)	Fellows (*n* = 190)
Self‐identified ethnicity		
Hispanic or LatinX	6 (10)	19 (10)
Self‐identified race		
Asian	5 (8.3)	28 (14.7)
Black or African American	0 (0)	10 (5.3)
White	50 (83.3)	142 (74.7)
Other	5 (8.3)	10 (5.3)
Self‐identified sex^a^		
Female	36 (60)	151 (79.5)
Prefer not to respond	1 (1.7)	2 (1.1)
MFM fellowship state		
Abortion restrictive	29 (49)	92 (48)
Abortion protective	30 (51)	98 (52)
MFM fellowship region		
Midwest	13 (22)	42 (22)
Northeast	15 (25)	57 (30)
South	18 (31)	54 (28)
West	13 (22)	37 (19)
Fellowship is affiliated with a CFP fellowship	19 (31.7)	61 (32.1)
MFM faculty engaged in abortion advocacy?	45 (75)	120 (63.2)
MFM faculty support fellow abortion advocacy?	100 [98.25, 100]	98 [75, 100]

*Note*: Other race includes American Indian/Alaska Native, Native Hawaiian or Other Pacific Islander, More Than One Race, Unknown/Not Reported. Values presented as median [P25, P75], *n* (%).

Abbreviations: CFP, complex family planning; MFM, maternal‐fetal medicine; PD, program director.

^a^ No responses for transgender female, transgender male, or gender nonconforming.

Overall, MFM PDs were significantly more likely to place importance on the ability for their fellows to have training in induction termination of fetuses with cardiac activity (92 [75, 100] vs. 72 [50, 99.5], *p* < 0.01) and induction of fetal asystole (90 [74.3, 100] vs. 73 [50, 98.5], *p* < 0.01, Table 2). There were no significant differences in perceived importance of D&C or D&E training between PDs and fellows, although both groups rated those as neutral or towards the more important end of the continuous scale (*p* > 0.05, Table 2). PDs and fellows generally shared similar views on the skillset they should possess for abortion‐related care after fellowship.

**TABLE 2:** PDs versus Fellows—Perspectives and goals for fellowship training.

	PDs (*n* = 60)	Fellows (*n* = 190)	Adjusted *p* value
In your opinion, how important is it for MFM fellows to access the following comprehensive reproductive health training?			
D&Cs	77 [50, 100]	67 [50, 96.3]	0.24
D&Es	77.5 [50, 100]	70 [50, 94.5]	0.34
Induction termination of fetuses with cardiac activity	92 [75, 100]	72 [50, 99.5]	**<0.01**
Induction of fetal asystole	90 [74.3, 100]	73 [50, 98.5]	**0.01**
Upon completing my MFM fellowship, I should be able to:			
Perform abortions (dilation and curettage, dilation and evacuation, and/or induction/termination)	80 [50, 99.3]	90 [65, 100]	0.34
Perform induction of fetal asystole	75 [50, 99]	81 [50, 100]	0.34
Work collaboratively with family planning specialists	100 [99, 100]	100 [94, 100]	0.34
Understand the local legal atmosphere to allow for provision of comprehensive reproductive services	100 [98, 100]	100 [87, 100]	0.34

*Note*: Values presented as median [P25, P75]. *p* values: Mann–Whitney for nonparametric variables, unpaired *t*‐test for parametric variables. Bold values suggest *p* < 0.05.

Abbreviations: D&C, dilation and curettage; D&E, dilation and evacuation; PD, program director.

Our secondary analysis comparing PDs to fellows based on state legislation on abortion care yielded notable differences in abortion‐restrictive states. In the adjusted analysis, there was no difference between PDs’ and fellows' in abortion‐protective states in their rating of importance of D&Cs, D&Es, induction termination of fetuses with cardiac activity, and induction of fetal asystole. In contrast, PDs in abortion‐restrictive states placed significantly more importance on ongoing training and exposure to D&Cs (82.5 [50, 100] vs. 50 [42.5, 74.5], *p* = 0.03) and induction termination of fetuses with cardiac activity (88.5 [68.8, 100] vs. 51 [50, 80], *p* = 0.01, Table 3) compared to fellows in those states. While there were no significant differences between PDs and fellows in abortion‐restrictive states on perceived importance of training in D&Es (77.5 [50, 100] vs. 50 [50, 80], *p* = 0.12) and induction of fetal asystole (80 [50, 98.3] vs. 50 [32, 79], *p* = 0.12), PDs trended towards more importance on those skills (Table 3). There were no differences in perceived needed skillset upon fellowship completion between PDs and fellows in either abortion‐protective or abortion‐restrictive states. A sensitivity analysis of respondents from abortion‐protective states, which excluded six fellow respondents from not‐protected states, did not result in notable differences.

**TABLE 3:** PDs versus Fellows—Perspectives and goals for fellowship training by state‐based legislation.

	Abortion‐protective states			Abortion‐restrictive states		
	PDs (*n* = 30)	Fellows (*n* = 98)	Adjusted *p* value	PDs (*n* = 30)	Fellows (*n* = 92)	Adjusted *p* value
In your opinion, how important is it for MFM fellows to access the following comprehensive reproductive health training?						
D&Cs	72 [50, 100]	75 [50, 100]	0.92	82.5 [50, 100]	50 [42.5, 74.5]	**0.03**
D&Es	77.5 [60.5, 100]	79.5 [50, 100]	0.92	77.5 [50, 100]	50 [50, 80]	0.12
Induction termination of fetuses with cardiac activity	95 [75, 100]	78.5 [50, 100]	0.51	88.5 [68.8, 100]	51 [50, 80]	**0.01**
Induction of fetal asystole	99.5 [80, 100]	85 [66.3, 100]	0.12	80 [50, 98.3]	50 [32, 79]	0.12
Upon completing my MFM fellowship, I should be able to:						
Perform abortions (dilation and curettage, dilation and evacuation, and/or induction/termination)	75 [50, 97.5]	92 [75, 100]	0.15	93 [55, 100]	82 [50, 100]	0.92
Perform induction of fetal asystole	80 [50, 100]	100 [75, 100]	0.20	64 [50, 93.5]	61 [33, 92]	0.92
Work collaboratively with family planning specialists	100 [100, 100]	100 [98.8, 100]	0.92	100 [85.5, 100]	100 [75, 100]	0.92
Understand the local legal atmosphere to allow for provision of comprehensive reproductive services	100 [99.8, 100]	100 [96.5, 100]	0.92	100 [92.75, 100]	100 [80.25, 100]	0.92

*Note*: Abortion‐protective states and territories: Those with Expanded/Protected/Not Protected legislation regarding abortion care include AK, CA, CO, CT, DC, DE, FL, HI, IL, KS, MA, MD, ME, MI, MT, NH, NJ, NM, NV, NY, OR, PR, RI, VA, VT, and WA. Abortion‐restrictive states and territories: Those with Hostile/Illegal legislation for abortion access include AL, AR, GA, Guam, IA, ID, IN, KY, LA, MI, MO, MS, NC, ND, NE, OH, OK, PA, SC, SD, TN, TX, UT, WI, WV, and WY. Values presented as median [P25, P75]. *p* values: Mann–Whitney for nonparametric variables, unpaired *t*‐test for parametric variables. Bold values suggest *p* < 0.05.

Abbreviations: D&C, dilation and curettage; D&E, dilation and evacuation; PD, program director.

Our analysis of fellows’ and PDs’ perspectives by state‐based abortion restrictions showed that fellows completing MFM fellowship in abortion‐protective states were significantly more likely to rate access to abortion‐related care as more important compared to those in abortion‐restrictive states (*p* < 0.01 for all, Table 4) [4]. Fellows in abortion‐protective states were more likely to place importance on knowing how to perform induction of fetal asystole (100 [75, 100] vs. 61 [33, 92], *p* = 0.001) and working collaboratively with CFP subspecialists (100 [98.8, 100] vs. 100 [75, 100], *p* = 0.02) with regard to necessary skills post‐fellowship (Table 4). However, PDs in both abortion‐protective and abortion‐restrictive states valued most abortion‐related training and competency in related skills post‐fellowship similarly (Table 4). While not statistically significant, PDs in abortion‐protective states trended toward placing higher importance on access to learning induction of fetal asystole (99.5 [80, 100] vs. 80 [50, 98.3], *p* = 0.06, Table 4). A sensitivity analysis for fellows again did not result in notable differences.

**TABLE 4:** Head‐to‐head comparison of fellows and PDs in abortion‐protective states versus abortion‐restrictive states.

	Fellows			PDs		
	Abortion protective (*n* = 98)	Abortion restrictive (*n* = 92)	Adjusted *p* value	Abortion protective (*n* = 30)	Abortion restrictive (*n* = 30)	Adjusted *p* value
In your opinion, how important is it for MFM fellows to access the following comprehensive reproductive health training?						
D&Cs	75 [50, 100]	50 [42.5, 74.5]	**0.001**	72 [50, 100]	82.5 [50, 100]	0.84
D&Es	79.5 [50, 100]	50 [50, 80]	**0.001**	77.5 [60.5, 100]	77.5 [50, 100]	0.84
Induction termination of fetuses with cardiac activity	78.5 [50, 100]	51 [50, 80]	**0.005**	95 [75, 100]	88.5 [68.8, 100]	0.84
Induction of fetal asystole	85 [66.3, 100]	50 [32, 79]	**0.001**	99.5 [80, 100]	80 [50, 98.3]	0.06
Upon completing my MFM fellowship, I should be able to:						
Perform abortions (dilation and curettage, dilation and evacuation, and/or induction/termination)	92 [75, 100]	82 [50, 100]	0.30	75 [50, 97.5]	93 [55, 100]	0.84
Perform induction of fetal asystole	100 [75, 100]	61 [33, 92]	**0.001**	80 [50, 100]	64 [50, 93.5]	0.63
Work collaboratively with family planning specialists	100 [98.8, 100]	100 [75, 100]	**0.02**	100 [100, 100]	100 [85.5, 100]	0.36
Understand the local legal atmosphere to allow for provision of comprehensive reproductive services	100 [96.5, 100]	100 [80.3, 100]	0.18	100 [99.75, 100]	100 [92.8, 100]	0.47

*Note*: Abortion‐protective states and territories: Those with Expanded/Protected/Not Protected legislation regarding abortion care include AK, CA, CO, CT, DC, DE, FL, HI, IL, KS, MA, MD, ME, MI, MT, NH, NJ, NM, NV, NY, OR, PR, RI, VA, VT, and WA. Abortion‐restrictive states and territories: Those with Hostile/Illegal legislation for abortion access include AL, AR, GA, Guam, IA, ID, IN, KY, LA, MI, MO, MS, NC, ND, NE, OH, OK, PA, SC, SD, TN, TX, UT, WI, WV, and WY. Values presented as median [P25, P75]. *p* values: Mann–Whitney for nonparametric variables, unpaired *T*‐test for parametric variables. Bold values suggest *p* < 0.05.

Abbreviations: D&C, dilation and curettage; D&E, dilation and evacuation; PD, program director.

## 4 DISCUSSION

Our data indicate that, in the current environment, fellows from abortion‐restrictive states placed lower importance on most abortion‐related training during fellowship than their PDs. In abortion‐protective states, there were no differences in importance rated by PDs and fellows regarding abortion‐related training and competency in these skills post‐fellowship. Fellows in abortion‐protective states consistently valued all surveyed skills more than those in abortion‐restrictive states, while PDs in abortion‐protective states rated all skills as similarly important.

There has been a general increase in abortion‐related interest among both MFM fellows and PDs over time [4, 8, 9, 10, 11]. However, our main findings contrast with a 2018 survey showing that MFM fellows expressed more interest in pregnancy counseling and D&E training than PDs [8]. A growing body of evidence suggests that although there is interest among MFM fellows to continue abortion related training in fellowship, the location of a fellowship program, and thus, the extent of abortion‐related training that is accessible, is related to an applicant's desire to obtain abortion training in fellowship [4, 11]. Whether location leads to a decreased perceived importance of these skills within MFM care or if decreased interest causes fellows to choose fellowships in abortion‐restrictive states cannot be determined from our data. It is also unclear why PDs, overall and particularly in abortion‐restrictive states, place more importance on abortion‐related care compared to fellows, although one can theorize that past lived experiences during times of fewer abortion restrictions, experiences leading trainees, and the increased sub‐specialization of MFM physicians all play a role. Fellows in abortion‐protective states overwhelmingly valued abortion‐related training and skills more than fellows in abortion‐restrictive states. They had similar responses to PDs across all regions, which suggests that the location and training environment are critical to the perceived importance of abortion‐related training in a fellowship.

Notably, fellows in abortion‐restrictive states placed lower importance on all abortion‐related training compared to their counterparts in abortion‐protective states (*p* < 0.05 for all), yet there was no significance in the skillset they thought was needed with regards to performing abortion care after fellowship (Table 4). This trend was also demonstrated when comparing PDs and fellows in abortion‐restrictive states (Table 3). It is unclear why there is this contrast between what fellows believe fellowships training should include and what they expect to be able to do at the end of their training. It is possible they may have had previous training during residency that they feel is sufficient to carry them forward, though it has been shown that fellows training in abortion‐restrictive states are significantly less likely to have had experience in D&Es and induction termination of fetuses with cardiac activity during residency compared to fellows training in abortion‐protective states [4]. Previous data suggest that MFM physicians who completed fellowship training at institutions with CFP programs were also more likely to perform abortions in their future practice [9]. However, it is also possible that the increasing sub‐specialization of care, especially in areas with access to CFP subspecialists, may lead trainees to perceive that abortion training is no longer primarily the responsibility of MFMs; instead, they may see it as the domain of a dedicated subspecialty.

Lastly, a notable discrepancy was observed between the high level of support for fellow advocacy reported by MFM faculty and the significantly lower perception of that support by the fellows. This divergence in perspectives on support for fellow advocacy between MFM faculty and fellows warrants further investigation to understand its potential impact on fellow well‐being, moral distress, and abortion‐related care [12]. It is plausible that, in restrictive environments, fellows may deprioritize abortion training if their role models are not actively supportive or engaged in advocacy work on abortion care.

Recent data provide conflicting evidence on whether the number of OB/GYNs practicing in states with abortion bans has decreased meaningfully compared to those in abortion‐protective states after the Dobbs decision [13, 14, 15]. These studies also do not evaluate the evolution of other aspects of reproductive health care, such as the moral distress felt by practitioners, the quality of training provided to residents and fellows, and the impact on reproductive health deserts [13, 14, 15, 16, 17]. While abortion‐related training is and should be one of the primary focuses of CFP fellowship, given the smaller number of CFP programs and how severely legal restrictions ultimately impact the recruitment of practitioners trained in these skills, there is not always a CFP‐trained subspecialist available [18]. Furthermore, based on a 2023 study of CFP fellows’ intended practice locations after fellowship training, only 50% of those who planned to go to an area with unmet needs did so [19]. Thus, in areas where there are not CFP‐trained providers and only MFMs, OB/GYN generalists, and family practitioners, the role of the MFM is to support their colleagues and have the skillset to provide abortion‐related care.

To ensure interested fellows are trained in the full scope of abortion‐related care, fellowship programs should secure access to abortion training in parallel with currently established ACGME MFM program requirements. At centers where abortion care is integrated into MFM fellowship, continued opportunities to participate should be protected. Institutions with coexisting MFM and CFP programs should consider strengthening collaboration and formalizing interdisciplinary training between the two fellowships [20]. For those who are training in locations without access to abortion‐related opportunities, formal external opportunities for training can also be arranged with national organizations, such as Planned Parenthood, clinical abortion training centers, or local community partners. Funding from external sources like grants or training programs through the Foundation for SMFM or Society of Family Planning should be promoted [21, 22]. These opportunities would allow MFM fellows to continue this education and training in abortion care simultaneously with other ACGME milestones.

One would be remiss not to acknowledge the limitations of our study. First, it is a cross‐sectional study. Although this survey was conducted during a time when some states faced increasing restrictions on abortion care, most of it took place before the *Dobbs* decision and before many trigger laws were implemented. As a result, our data do not capture any changes in attitudes among PDs and MFM fellows since that time. An additional inherent limitation of the survey is its susceptibility to selection bias, as respondents may represent the extremes of attitudes regarding these complex topics rather than being representative of all PDs and MFM fellows. Furthermore, it is possible that the use of a continuous sliding scale for assessment of attitudes may have been more difficult to understand than the typical 10‐point Likert scale. While the use of a continuous scale provides more precision than a typical 10‐point Likert scale and reduces categorization bias, it is possible that this interface created more complex usability issues. We chose not to analyze these data categorically to reduce transformation error, though it is possible that using the sliding scale set to a default of 50 (middle or neutral) is prone to starting bias. One of the main strengths of this survey is the even distribution of PDs and fellows training in both abortion‐protective and abortion‐restrictive states, as well as across the different regions of the United States, which suggests that these findings may be more generalizable. Our survey had a response rate of 61% for PDs and 46% for fellows, which is comparable to previous surveys administered to MFM fellows and providers published in peer‐reviewed journals [5, 8].

## 5 CONCLUSION

MFM subspecialists are in a unique position to provide abortion‐related reproductive care, especially in areas lacking advanced family planning training and reproductive care practitioners. However, our findings highlight that the difference in importance placed on these procedures in this subspecialty over time is creating not only a regional but also a generational divide on what is perceived to be within the scope of MFM practice.

## AUTHOR CONTRIBUTIONS

**CeCe Cheng**: Writing—review and editing; writing—original draft; visualization; resources; project administration; methodology; investigation; funding acquisition; data curation, conceptualization. **John J. Byrne**: Writing—review and editing; supervision; methodology; conceptualization. **Jennifer L. Kerns**: Writing—review and editing; supervision; methodology. **Patrick S. Ramsey**: Visualization; supervision; funding acquisition; conceptualization. **Ashish Premkumar**: Writing− review and editing; visualization; validation; supervision' methodology; investigation; conceptualization.

## CONFLICT OF INTEREST STATEMENT

The authors declare no conflicts of interests.
